# Functional Analysis of the “Green Revolution” Gene *Photoperiod-1* and Its Selection Trends During Bread Wheat Breeding

**DOI:** 10.3389/fpls.2021.745411

**Published:** 2021-11-11

**Authors:** Yongzhen Wu, Jiahui Liu, Guimei Hu, Huixian Xue, Huiyuan Xu, Chunhua Zhao, Ran Qin, Fa Cui, Han Sun

**Affiliations:** ^1^College of Agriculture, Ludong University, Yantai, China; ^2^Key Laboratory of Molecular Module-Based Breeding of High Yield and Abiotic Resistant Plants in Universities of Shandong, Ludong University, Yantai, China

**Keywords:** circadian clock, *Ppd-1*, molecular marker, haplotype, association analysis, yield-related traits, artificial selection, *Triticum aestivum*

## Abstract

Flowering is central to the transformation of plants from vegetative growth to reproductive growth. The circadian clock system enables plants to sense the changes in the external environment and to modify the growth and development process at an appropriate time. *Photoperiod-1* (*Ppd-1*), which is controlled by the output signal of the circadian clock, has played an important role in the wheat “Green Revolution.” In the current study, we systematically studied the relationship between *Ppd-1* haplotypes and both wheat yield- and quality-related traits, using genome-wide association analysis and transgenic strategies, and found that highly appropriate haplotypes had been selected in the wheat breeding programs. Genome-wide association analysis showed that *Ppd-1* is associated with significant differences in yield-related traits in wheat, including spike length (SL), heading date (HD), plant height (PH), and thousand-grain weight (TGW). *Ppd-1-Hapl-*A1 showed increased SL by 4.72–5.93%, whereas *Ppd-1-Hapl-*B1 and *Ppd-1-Hapl-D1* displayed earlier HD by 0.58–0.75 and 1.24–2.93%, respectively, decreased PH by 5.64–13.08 and 13.62–27.30%, respectively, and increased TGW by 4.89–10.94 and 11.12–21.45%, respectively. Furthermore, the constitutive expression of the *Ppd-D1* gene in rice significantly delayed heading date and resulted in reduced plant height, thousand-grain weight, grain width (GW), and total protein content. With reference to 40years of data from Chinese wheat breeding, it was found that the appropriate haplotypes *Ppd-1-Hapl-*A1, *Ppd-1-Hapl*-B1, and *Ppd-1-Hapl*-D1 had all been subjected to directional selection, and that their distribution frequencies had increased from 26.09, 60.00, and 52.00% in landraces to 42.55, 93.62, and 96.23% in wheat cultivars developed in the 2010s. A *Ppd-B1* methylation molecular marker was also developed to assist molecular wheat breeding. This research is of significance for fully exploring the function of the *Ppd-1* gene and its genetic resource diversity, to effectively use the most appropriate haplotypes and to improve crop yield and sustainability.

## Introduction

Flowering is the central process in plant transformation from vegetative growth to reproductive growth, and photoperiod is one of the key environmental factors that regulates this process. In the photoperiod pathway which controls flowering in higher plants, the plant circadian system is at a key position and plays an important role in regulating the flowering of plants. During the long-term evolution of plants, the circadian system gave plants the ability to adapt to periodic changes in the external environment. The circadian system allows plants to sense changes in the external environment and to complete the growth and development process at the appropriate time (Greenham and McClung, 2015; Wei et al., 2018). The circadian system includes inputs from external signals and internal core oscillator and output channels (Harmer, 2009). Certain biological processes regulated by the circadian clock will also feedback and regulate the core oscillator of the circadian clock, forming a complex feedback regulatory network (Nohales and Kay, 2016).

The molecular mechanism of the circadian clock of the model plant *Arabidopsis* has been studied in depth. The *Arabidopsis* circadian clock includes several feedback loops. The central feedback loop is composed of genes encoding the core oscillator members *TIMING OF CAB EXPRESSION 1* (*TOC1*), *LATE ELONGATED HYPOCOTYL* (*LHY*), and *CIRCADIAN CLOCK ASSOCIATED 1* (*CCA1*; Alabadí et al., 2001). *CCA1*/*LHY* also forms an early feedback loop with *PRR9*/*PRR7* of the *PSEUDO RESPONSE REGULATOR* (*PRR*) gene family. *PRR9*, *PRR7*, and *PRR5* exhibit expression peaks in sequence every 2–3h from dawn to evening. They recruit histone deacetylases to form a transcriptional repression complex, which inhibits the expression of *CCA1* and *LHY* at the transcriptional level (Nakamichi et al., 2010; Wang et al., 2013). The late feedback loop contains the evening complex (EC; Nusinow et al., 2011), composed of MYB transcription factor LUX ARRHYTHMO, nuclear protein EARLY FLOWERING 3 (ELF3), ELF4 (Nusinow et al., 2011), and TOC1. These feedback loops interlock to form the basic structure of the repressilator of the core oscillator of the circadian clock.

The *PRR* gene family, the core component of the circadian clock, has an important function in crops. The “Green Revolution” gene in wheat, *Ppd-1* (*TaPRR37*), encodes a member of the *PRR* protein family, is homologous to *Arabidopsis* PRR7, and includes three photoperiod response loci, namely *Ppd-A1*, *Ppd-B1*, and *Ppd-D1* (Laurie, 1997; Worland and Snape, 2001; Beales et al., 2007). The deletion of the *Ppd-A1* promoter region is related to photoperiod insensitivity. Nishida et al. (2013) found that the promoter region of common wheat “Chihokukomugi” has a 1,085bp deletion. Wilhelm et al. (2009) studied near-isogenic lines of tetraploid durum wheat with different photoperiod responses and found that the photoperiod insensitivity was related to two independent deletions (1,027bp deletion and 1,117bp deletion, respectively) in the *Ppd-A1* gene, which caused abnormal gene expression and activation of *FLOWERING LOCUS T* (*FT*) expression. The aforementioned *Ppd-A1* promoter deletion variant alleles were named *Ppd-A1a.1* (1,085bp deletion; Nishida et al., 2013), *Ppd-A1a.2* (1,027bp deletion), and *Ppd-A1a.3* (1,117bp deletion; Wilhelm et al., 2009), respectively. The photoperiod-insensitive allele *Ppd-A1a* confers wheat with a photoperiod-insensitive phenotype, which is intermediate between the insensitive phenotypes caused by the alleles *Ppd-B1a* and *Ppd-D1a* (Bentley et al., 2011; Shaw et al., 2012). Muterko et al. (2015) reported the allelic variation of the photoperiod-sensitive site *Ppd-A1b*. According to the difference in the movement speed of the 452bp fragment in the promoter region, *Ppd-A1b* can be divided into two allele types: *Ppd-A1b.AI* and *Ppd-A1b.AII*.

Studies have shown that the *Ppd-B1a* photoperiod-insensitive allele is caused by copy number variation, with increased copy number leading to increased gene expression levels, and achieving a photoperiod-insensitive phenotype (Díaz et al., 2012). According to the types of *Ppd-B1* copy number, it can be divided into *Ppd-B1a* (three-copy), *Ppd-B1b* (one-copy), *Ppd-B1c* (four-copy), *Ppd-B1d* (two-copy), and *Ppd-B1e* (null allele; Díaz et al., 2012; Cane et al., 2013). Würschum et al. (2015) studied the distribution characteristics of *Ppd-B1* copy number in 1,110 winter wheat accessions and the effects of copy number on flowering time. The results showed that copy number variation in *Ppd-B1* facilitated global adaptation in wheat. Sun et al. (2014) proved that the level of DNA methylation in the promoter region of the *Ppd-B1* gene affected gene expression and was associated with photoperiod insensitivity. According to the levels of DNA methylation, it can be divided into two types: *Ppd-B1* methylation haplotype a and methylation haplotype b (Sun et al., 2014). It is worth noting that DNA hypermethylation at *Ppd-B1a* is accompanied by higher copy numbers, either of which effects might be factors affecting the development of the *Ppd-B1a* allele (Sun et al., 2014).

Beales et al. (2007) showed that wheat accessions carrying the photoperiod-insensitive allele *Ppd-D1a* all contained a 2kb deletion upstream of the coding region. The deletion caused abnormal expression of the *Ppd-D1* gene, leading to the expression of *FT* under short-day conditions. According to the promoter 2kb deletion and other allelic variants (including TE insertion of the first intron and 5bp deletion of the seventh exon), *Ppd-D1* can be divided into four allele types: *Ppd-D1a*, *Ppd-D1b*, *Ppd-D1c*, and *Ppd-D1d*, of which only *Ppd-D1a* contains a 2kb deletion (Beales et al., 2007; Guo et al., 2010; Cane et al., 2013). In addition to regulating the photoperiod response of wheat, *Ppd-1* is also a key regulator of inflorescence architecture and paired spikelet development (Boden et al., 2015).

Although there has been considerable research into the factors underlying the formation of *Ppd-1* photoperiod-insensitive alleles, studies on the development of molecular markers for *Ppd-1* genetic and epigenetic variation, the relationships between *Ppd-1* haplotypes and yield-related traits, grain characteristics and quality traits on a genome-wide scale, and the effects of selection on *Ppd-1* haplotypes in wheat breeding programs are incomplete. In the current study, we conducted a systematic functional analysis of *Ppd-1*, using genome-wide association analysis and studies on transgenics, and explored the relationship between *Ppd-1* alleles and yield- and quality-related traits of wheat. Using data from 40years of wheat breeding in China, the inadvertent effects of selection for increased yield and improved quality on *Ppd-1* haplotype were systematically explored. As a consequence, this study provides a theoretical basis for revealing the function of *Ppd-1* and identifying its application to wheat breeding.

## Materials and Methods

### Plant Material

A total of 188 wheat accessions, derived from the major agroecological wheat regions of China and consisting of 25 landraces and 163 modern cultivars, was used for genome-wide association analysis and breeding selection analysis (Supplementary Table S1). In the population of modern cultivars, 9, 26, 75, and 53 accessions were released in the 1980s, 1990s, 2000s, and 2010s, respectively. The accessions were separately planted in Qixia (121.07°E, 37.49°N), Weifang (119.44°E, 36.68°N), Pulagu (121.41°E, 37.31°N), Shijiazhuang (114.69°E, 37.89°N), and Ludong University (121.35°E, 37.51°N) during the years 2017–2020 (Supplementary Table S2). The test locations are all located in the northern part of China, with long-day conditions. Each accession was planted in 2m three-row plots with 30cm between rows. At maturity, six plants in the middle of each plot were selected for each genotype in order to investigate agronomic traits, including plant height (PH), spike length (SL), spike number (SN), total number of spikelets per spike (TNSS), number of grains per spike (NGS), thousand-grain weight (TGW), heading date (HD), grain length (GL), and grain width (GW). PH was measured from the stem base to the top of the main tiller spike. SL was measured from the internodes to the spike tip (excluding awns). TNSS and NGS were measured from the main tiller spike. HD was recorded on 50% spike emergence. TGW, GL, and GW were determined using the Intelligent Test and Analysis System (TOP Cloud-agri Technology, Zhejiang, China) using seeds after harvesting.

### *Ppd-B1* Methylation-Sensitive Restriction Endonuclease Marker Development and *Ppd-1* Functional Molecular Markers

Based on the *Ppd-B1* differentially methylated region, a target fragment (−1,250 to −665), containing methylation-sensitive restriction endonuclease (MSRE) *Hpa*II or *BstU*I recognition sites (CCGG or CGCG), was selected to develop MSRE markers. DNA samples were extracted from 7-days-old seedlings during the light period using a phenol–chloroform method (Sharp et al., 1988). When detecting the methylation level of the target material, *Hpa*II or *BstU*I was first used to digest the genomic DNA, and then, the digested product was amplified with the primers B1-HpaII-F1/R1 (Supplementary Table S3). The methylation type of *Ppd-B1* can be distinguished according to the presence or absence of the target fragment after electrophoresis on 1.5% agarose gel. Amplification of the target band indicates that the identified material is the *Ppd-B1* hypermethylated type, whereas the absence of an amplification product indicates that the identified material is of the *Ppd-B1* hypomethylated type.

The promoter region of *Ppd-A1b* was amplified using previously published primers: durum_Ag5del_F2/durum_Ag5del_R2 (Wilhelm et al., 2009). Amplification products were separated on 6.5% nondenaturing polyacrylamide (PAA) gels. Separation of PCR amplicons by PAA gel electrophoresis revealed differences in the migration rate for the 452bp fragments: AI (slow-migrating 452bp fragments, 452s) and AII (fast-migrating 452bp fragments, 452f; Muterko et al., 2015).

Primers S64-copy-F1/S64-copy-R1 were used to identify the “Sonora64”-type *Ppd-B1a* allele (three-copy). Primers CS-copy-F1/CS-copy-R1 were used to identify the “Chinese Spring”-type *Ppd-B1c* allele (four-copy). The *Ppd-B1b* (one-copy) and *Ppd-B1d* (two-copy) alleles were identified using quantitative analysis with primers CNV10-F/CNV10-R and C-F/C-R (Díaz et al., 2012; Cane et al., 2013).

The 2kb deletion in the promoter region of *Ppd-D1a* allele was amplified using a common forward primer Ppd-D1_F combined with two reverse primers, Ppd-D1_R1 and Ppd-D1_R2. Markers D78 and D520 were used to detect the insertion of TE in the first intron. Marker D5 was used to detect the 5bp deletion in the seventh exon. This assay used nested PCR with two pairs of primers, D5-1F/D5-1R and D5-2F/D5-2R. Primers exon8_F1/exon8_R1 were used to detect the 16bp insertion in the eighth exon (Beales et al., 2007; Guo et al., 2010). All *Ppd-1* functional molecular markers are listed in Supplementary Table S3.

### Bisulfite Genomic Sequencing

Hexaploid wheats “Am3,” “Laizhou953,” “Chinese Spring,” “Lumai14,” and “Yanzhan1” were used for bisulfite genomic sequencing. Bisulfite conversion of genomic DNA was achieved using the EZ DNA Methylation-Gold™ Kit (Zymo Research, Irvine, CA, United States). The PCR products were purified and cloned into the pEASY-T1 Cloning Vector (TransGen, Beijing, China). At least 8–10 individual clones were sequenced, and three biologically independent replicates were carried out on each genotype to determine the methylation status of the target genomic regions. The sequencing data were analyzed by Kismeth software (Gruntman et al., 2008). The primer used for bisulfite genomic sequencing was referred to Sun et al. (2014; Supplementary Table S4).

### Reverse Transcription PCR and Quantitative Real-Time PCR

“Chinese Spring” was used to analyze the expression of *Ppd-A1*, *Ppd-B1*, and *Ppd-D1* in various organs during wheat development. The materials were placed in a vernalization incubator (16h light/8h dark, 5°C) for 15days and then transferred to a controlled environment room under LD conditions (16h light/8h dark, 24°C). “Chinese Spring” tissue samples included the shoot, leaf, leaf sheath, tiller base, flag leaf, pulvinus, young ear, and grain, which were collected at the seedling stage, three-leaf, tillering, flag leaf, full boot, ear emergence, anthesis, and milk grain stages, respectively. Four plants were mixed at each time point, and three biologically independent replications were performed for each tissue sample. Total RNA was extracted using RNAiso Plus (Takara, Ohtsu, Shiga, Japan). DNA was removed by digestion with DNase I (Fermentas, Ontario, Canada), and first-strand cDNA was synthesized using Moloney Murine Leukemia Virus (M-MLV) reverse transcriptase (Invitrogen, CA, United States). The cDNA was diluted 5-fold for reverse transcription PCR (RT-PCR). The amplified products were detected following gel electrophoresis on 2% agarose. The cDNA was diluted 10-fold for quantitative real-time PCR (qPCR). Reactions included 10μl 2×TB Green *Premix Ex Taq II* Mix, 0.8μl forward primer, 0.8μl reverse primer, and 2μl cDNA template in a total volume of 20μl. Reaction conditions were [95°C 30s; (95°C 5s, 60°C 34s)×40cycles], followed by a melting curve with 0.2°C steps between 60 and 95°C. qPCR was conducted using SYBR® Premix Ex *Taq*™ (Takara) on an ABI PRISM 7500 bio-analyzer (Applied Biosystems, Foster City, CA, United States). Fluorescence threshold is set in the exponential phase, and the Ct value (the cycle value at which each sample reached the fluorescence threshold) was extracted for each sample. Expression levels of *Ppd-A1*, *Ppd-B1*, and *Ppd-D1* genes were standardized against that of the housekeeping gene *glyceraldehyde-3-phosphate dehydrogenase* (*GAPDH*), and quantitative data were normalized using the 2^-ΔΔCt^ method (Livak and Schmittgen, 2001). All primers used in this study were designed using Primer Premier 5.0 software (Supplementary Table S4).

### Genome-Wide Associations and Population Structure Analysis

The 55K single nucleotide polymorphism (SNP) genotyping assay was filtered in PLINK1.9 “--maf 0.01 --geno 0.2 --mind 0.2.” A total of 24,904 unique markers was analyzed for genome-wide associations using the mixed linear model (PCA+K) method by TASSEL v5.2.72 (Bradbury et al., 2007), which took the population structure and relative kinship into account. A linkage disequilibrium heatmap was constructed using the package LDBlockShow v1.40 (Dong et al., 2020). Q–Q plots were generated using R package “rMVP” (Yin et al., 2021). Data for different phenotypic traits were represented by boxplots using the best linear unbiased estimate (BLUE) mean under each environment and were conducted using the R package “lme4” (Douglas et al., 2015).

The population structure of the 188 accessions was assessed with a subset of 3,912 markers using ADMIXTURE v1.3.0 (Alexander et al., 2009). The subset was selected using PLINK1.9 “--maf 0.01 --geno 0.2 --mind 0.2 --indep-pairwise 1,000 10 0.4.” The R package “pophelper” was used to generate the ancestry bar plots.

### Vector Construction, Plant Transformation, and Trait Measurements

The full-length open reading frame of *Ppd-D1* was amplified and inserted into the binary vector pCAMBIA1301 (Sun et al., 2021), using the homologous recombination method. The p*Ubi:Ppd-D1* construct was transferred into the *japonica* rice cultivar Zhonghua 17 (ZH17) by *Agrobacterium*-mediated transformation. Phenotypic measurements of the positive transgenic plants were performed using three independent transgenic lines (10–20 individuals per line) in Ludong University (121.35°E, 37.51°N). Ludong University is located in the northern part of China, with long-day conditions. Yield-related traits assessed included heading date (HD), PH, panicle length, number of grains per main panicle, TGW, and tiller number. Grain-related traits, including grain length (GL), grain width (GW), grain length/width ratio, and grain area, were determined using the Intelligent Test and Analysis System (TOP Cloud-agri Technology, Zhejiang, China). Grain quality traits, namely total protein content, total starch content, total amylose content, and total lipid content, were determined, with the total protein content and total lipid content of each grain sample being measured as described previously (Bradford, 1976; de Castro and Priego-Capote, 2010), whereas the total starch and amylose contents were determined with specific kits in accordance with the manufacturer’s instructions (Megazyme, County Wicklow, Ireland).

### Statistical Analysis

All statistical tests were performed using SPSS Statistics 18.0 (IBM, Armonk, NY, United States). Tukey’s multiple comparison test was used to determine statistical differences identified by one-way ANOVA. Significance was accepted at *p*<0.05 (*) and *p*<0.01 (**) levels.

## Results

### Methylation Molecular Marker Development and Expression Analysis of *Ppd-1*

**Figure 1:**
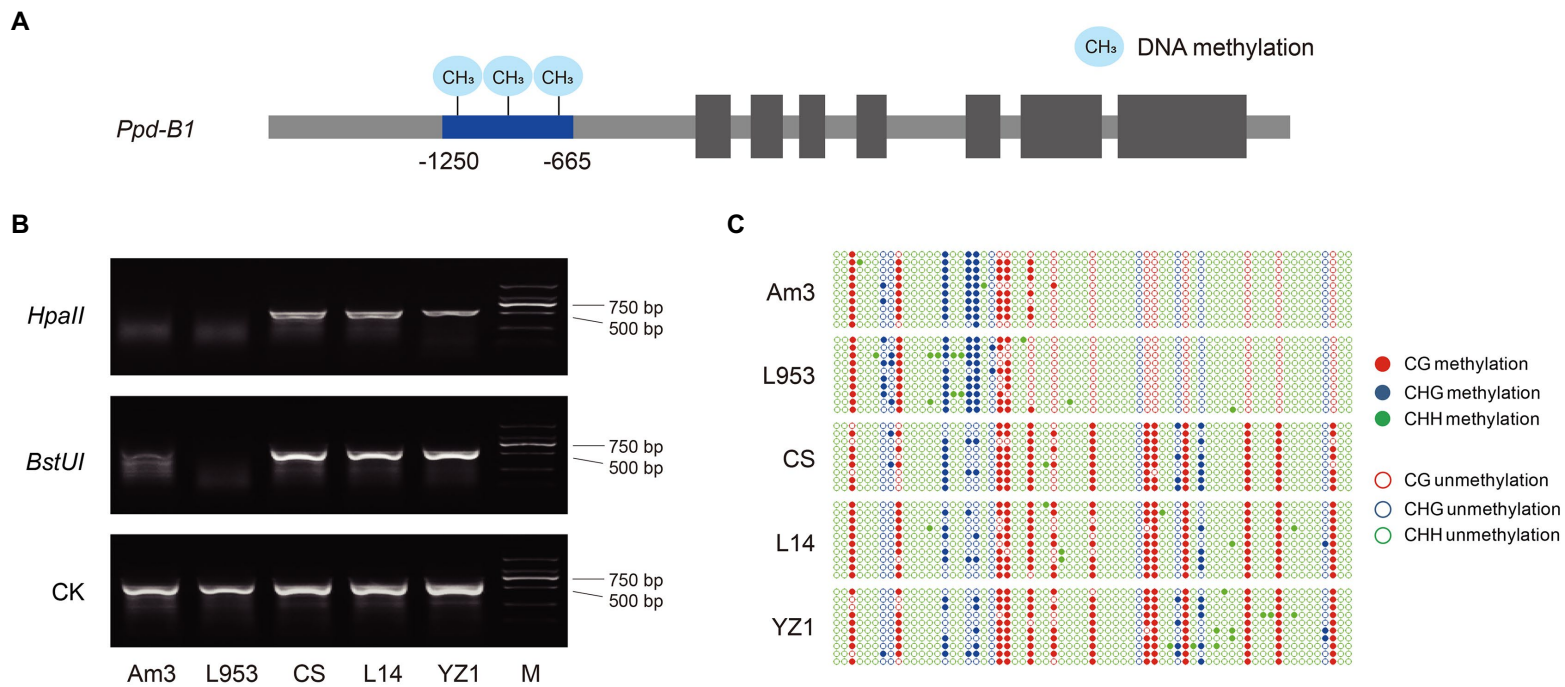
Development and verification of *Ppd-B1* methylation molecular markers. **(A)** *Ppd-B1* differential methylation region (−1,250 to −665), which contains the methylation-sensitive restriction endonuclease (MSRE) *Hpa*II or *BstU*I recognition sites (CCGG or CGCG), was selected to design markers. **(B)** A MSRE marker was used to identify wheat accessions with different methylation levels. Undigested DNA is shown as a control. **(C)** Materials with different *Ppd-B1* methylation levels were identified by the bisulfite genomic sequencing method. The solid and hollow circles represent methylated and unmethylated cytosine residues, respectively. Red, blue, and green circles represent CG, CHG, and CHH contexts, respectively. Data shown are representative of the results of three independent biological replicates. The accession abbreviations are L953, “Laizhou 953”; CS, “Chinese Spring”; L14, “Lumai 14”; and YZ1, “Yanzhan 1.”

The methylation levels of the *Ppd-B1* promoter have been shown to differ among wheat varieties (Sun et al., 2014). A MSRE marker was developed in the present study to detect the methylation of *Ppd-B1*. The promoter region (−1,250 to −665), containing methylation-sensitive restriction endonuclease *Hpa*II or *BstU*I recognition sites (CCGG or CGCG), was selected to develop the MSRE marker (Figure 1A). This MSRE marker could distinguish the *Ppd-B1* methylation level based on the restriction band type (Figure 1B), which was consistent with the bisulfite sequencing result (Figure 1C). Furthermore, molecular markers detecting *Ppd-A1* promoter variation (Wilhelm et al., 2009; Muterko et al., 2015), *Ppd-B1* copy number variation (Díaz et al., 2012; Cane et al., 2013), and *Ppd-D1* genetic variation (Beales et al., 2007; Guo et al., 2010) were all derived from published literature (Supplementary Table S3).

**Figure 2:**
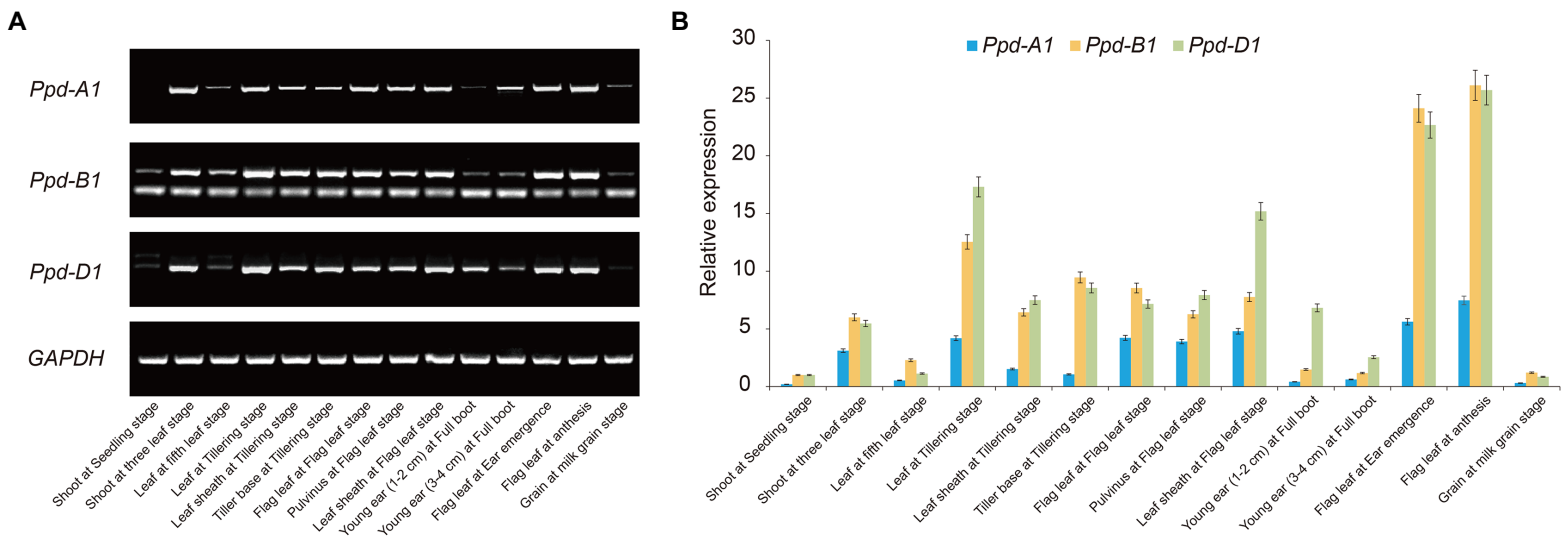
Expression patterns of *Photoperiod-1* (*Ppd-1*) in different organs at different developmental stages of hexaploid wheat “Chinese Spring” under LD conditions. **(A)** Expression of *Ppd-A1*, *Ppd-B1*, and *Ppd-D1* was analyzed by reverse transcription PCR (RT-PCR). Total RNAs were isolated from various organs of hexaploid wheat “Chinese Spring” at the seedling stage, three-leaf stage, tillering stage, flag leaf stage, full boot stage, ear emergence stage, anthesis, and milk grain stage. **(B)** Expression of *Ppd-A1*, *Ppd-B1*, and *Ppd-D1* was analyzed by quantitative real-time PCR (qPCR). The *glyceraldehyde-3-phosphate dehydrogenase* (*GAPDH*) gene was used as an endogenous control. The data are the means of three independent replicates.

The expression patterns of the *Ppd-1* genes were analyzed in various organs of “Chinese Spring” during wheat development under LD conditions (Figure 2). Overall, *Ppd-A1*, *Ppd-B1*, and *Ppd-D1* exhibited similar expression characteristics. They all showed relatively high expression levels during the tillering, flag leaf, ear emergence, and anthesis stages, whereas the expression level of *Ppd-1* was relatively low at the seedling, full boot, and milk grain stages. Moreover, *Ppd-1* expression in the flag leaf was higher than that in the young ear and the grain. It is worth noting that the expression level of *Ppd-A1* was significantly lower than that of the *Ppd-B1* and *Ppd-D1* genes during wheat development.

### Genome-Wide Association Analysis of *Ppd-1* With Agronomic Traits

**Figure 3:**
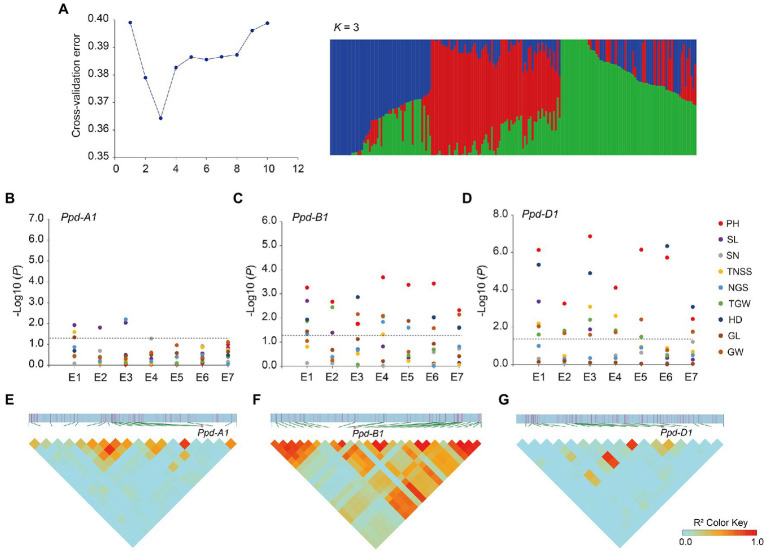
Population structure, association analysis, and linkage disequilibrium analysis. **(A)** Population structure of the natural population. Plot of cross-validation (CV) error against putative *k* ranging from 1 to 10 (left). Stacked bar plot of ancestry relationship of 188 accessions when *k*=3 (right). **(B)** Genome-wide association analysis of *Ppd-A1*. **(C)** Genome-wide association analysis of *Ppd-B1*. **(D)** Genome-wide association analysis of *Ppd-D1*. PH, plant height; SL, spike length; SN, spike number; TNSS, total number of spikelets per spike; NGS, number of grains per spike; TGW, thousand-grain weight; HD, heading date; GL, grain length; and GW, grain width. E1–E7 indicate the environments. Negative log_10_-transformed *p* values are plotted. The black horizontal dotted line indicates the threshold value for significant associations (*p*<0.05). **(E)** Linkage disequilibrium analysis spanning the physical position from 36.627 to 46.152Mb of chromosome 2A. **(F)** Linkage disequilibrium analysis spanning the physical position from 58.783 to 67.298Mb of chromosome 2B. **(G)** Linkage disequilibrium analysis spanning the physical position from 31.801 to 40.775Mb of chromosome 2D. The intensity of red shading indicates the level of linkage disequilibrium (*r*^2^) between variants.

Haplotype analysis of *Ppd-1* was performed on the population of 188 wheat accessions (Supplementary Table S1), using the *Ppd-1* molecular markers (Supplementary Table S3), and the basis of haplotype classification is shown in Supplementary Table S5. According to the classification of functional alleles, *Ppd-A1* can be divided into two haplotypes, *Hapl*-A1 and *Hapl*-A2, and the corresponding alleles are *Ppd-A1b.AI* and *Ppd-A1b.AII*, respectively (Wilhelm et al., 2009; Muterko et al., 2015). *Ppd-B1* is divided into two haplotypes, *Hapl*-B1 and *Hapl*-B2, according to the copy number (Díaz et al., 2012) and DNA methylation level (Sun et al., 2014). The alleles corresponding to *Hapl*-B1 are *Ppd-B1a*, *Ppd-B1c*, and *Ppd-B1d*, and the allele corresponding to *Hapl*-B2 is *Ppd-B1b* (Cane et al., 2013). *Ppd-D1* is divided into two haplotypes *Hapl*-D1 (*Ppd-D1a*) and *Hapl*-D2 (*Ppd-D1b*, *Ppd-D1c*, and *Ppd-D1d*) according to whether the promoter region has a 2kb fragment deletion (Beales et al., 2007; Guo et al., 2010; Cane et al., 2013). ADMIXTURE software was used to analyze the natural population structure. The results showed that the cross-validation error (CV error) value was lowest when *k* (number of subpopulation)=3, indicating that the population material could be divided into three subgroups (Figure 3A).

In order to investigate the relationship between *Ppd-1* haplotype and yield-related traits, we performed an association analysis of each haplotype with nine yield traits (PH; SL; SN; TNSS; NGS; TGW; HD; GL; and GW). The different accessions were each planted in trial sites at Qixia (121.07°E, 37.49°N), Weifang (119.44°E, 36.68°N), Pulagu (121.41°E, 37.31°N), Shijiazhuang (114.69°E, 37.89°N), and Ludong University (121.35°E, 37.51°N) during the years 2017–2020 (Supplementary Table S2).

Based on the *Ppd-1* genotyping data, combined with the Wheat 55K SNP array of the natural population, a genome-wide association analysis was performed. Association analysis showed the homeolog-specific functions of *Ppd-1*. For *Ppd-A1*, there were weak associations between *Ppd-A1* and spike length (three environments; Figure 3B). However, *Ppd-B1* was significantly associated with plant height (seven environments) and heading date (four environments), and weakly associated with thousand-grain weight (four environments) and grain width (four environments; Figure 3C). Association analysis showed that *Ppd-D1* had the strongest effect. Similar to *Ppd-B1*, *Ppd-D1* was strongly associated with plant height and heading date in all environments and moderately associated with thousand-grain weight and grain width (six environments; Figure 3D).

Using Wheat 55K SNP array data, linkage disequilibrium analysis was performed, spanning 5-Mb regions upstream and downstream of *Ppd-A1*, *Ppd-B1*, and *Ppd-D1*. The results showed that, for *Ppd-A1*, 19 SNP markers spanned the physical position from 36.627 to 46.152Mb (IWGSC RefSeq v2.1), which exhibited weak linkage disequilibrium (*r*^2^<0.5; Figure 3E), whereas *Ppd-B1* was in strong linkage disequilibrium (*r*^2^>0.5) with other significant variants, creating a linkage disequilibrium block spanning 58.783–67.298Mb (Figure 3F). For *Ppd-D1*, 18 SNP markers spanned the physical position from 31.801 to 40.775Mb, exhibiting weak linkage disequilibrium (*r*^2^<0.5; Figure 3G).

### Phenotypic Variation in *Ppd-1* Haplotypes

**Figure 4:**
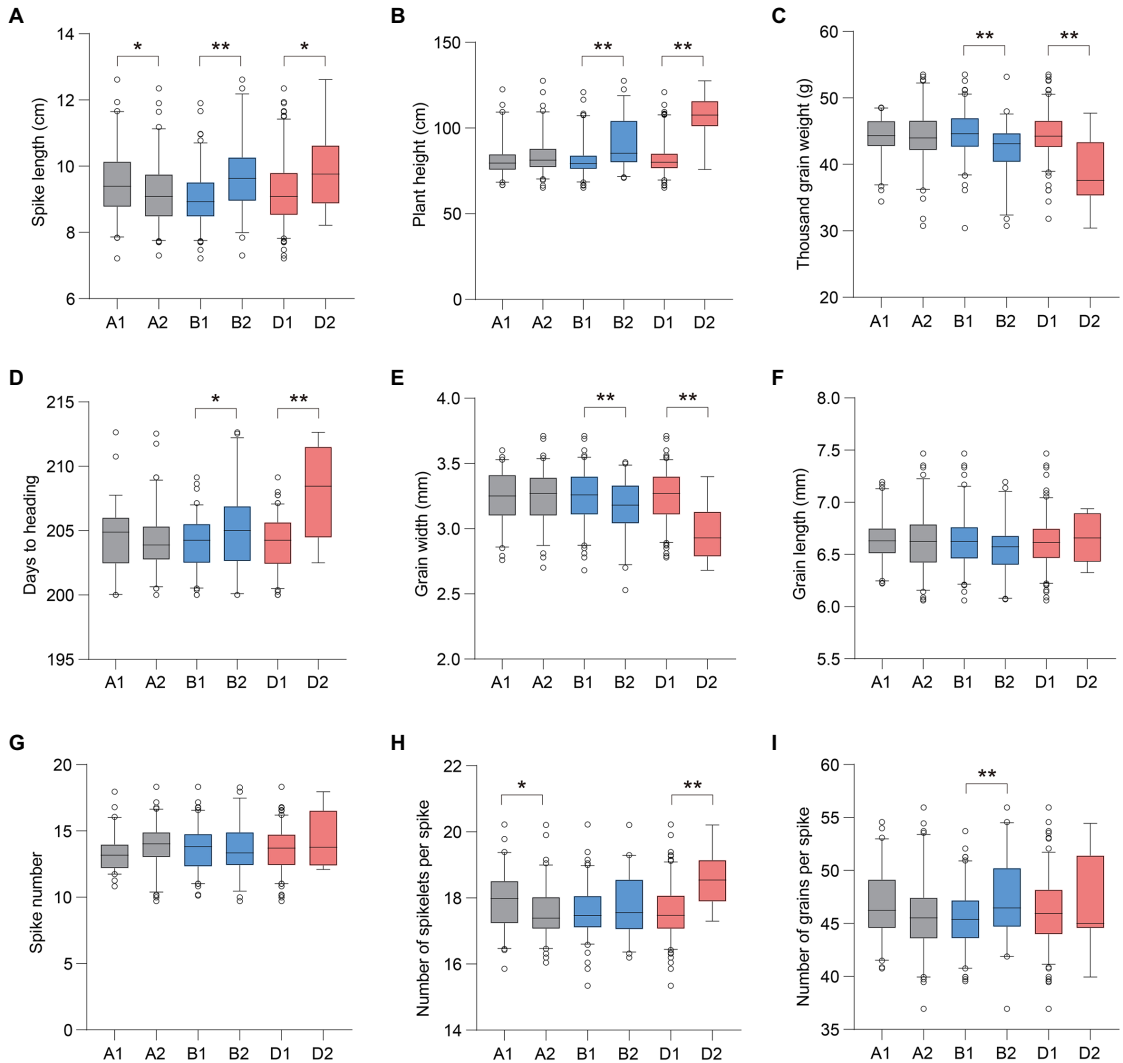
Phenotypic comparisons of large-effect haplotypes of *Ppd-1* homeologs. The phenotypic values of SL, PH, TGW, days to heading, GW, GL, SN, NGS, and number of grains per spike of different *Ppd-1* haplotypes are shown in **(A–I)**, respectively. A total of 188 wheat accessions were used for phenotypic data analysis. The accessions were separately planted in Qixia (121.07°E, 37.49°N), Weifang (119.44°E, 36.68°N), Pulagu (121.41°E, 37.31°N), Shijiazhuang (114.69°E, 37.89°N), and Ludong University (121.35°E, 37.51°N) during the years 2017–2020. The test locations are all located in the northern part of China, with long-day conditions. The best linear unbiased estimate (BLUE) mean of two replicates under each environment was estimated using mixed liner model by regarding accession as fixed effect and replicates as random effect. Data for different phenotypic traits are represented by boxplots using the BLUE mean under each environment. The horizontal solid line represents the median. The upper and lower edges of the box represent the upper and lower quartiles, respectively. The short thin lines represent the maximum and minimum values. The circle represents the outliers. ^*^p<0.05, ^**^p<0.01.

We compared the phenotype variations in the above agronomic traits associated with different *Ppd-1* haplotypes. In general, for *Ppd-A1*, accessions with *Hapl*-A1 showed a longer SL than *Hapl*-A2 (Figure 4A). For *Ppd-B1* and *Ppd-D1*, accessions with *Hapl*-B1 and *Hapl*-D1 showed more favorable phenotypic traits, including reduced PH, higher TGW, and earlier HD (Figures 4B–D). Specifically for *Ppd-A1*, the SL of *Hapl*-A1 increased by about 0.42–0.49cm, with an increase of 4.72–5.93%, compared with *Hapl*-A2. For *Ppd-B1*, the HD of *Hapl*-B1 was about 1.23–1.50days earlier, with an advance range of 0.58–0.75%. *Hapl*-B1 reduced PH by 5.00–10.97cm, with a decrease of 5.64–13.08% compared with *Hapl*-B2. *Hapl*-B1 increased TGW by about 1.75–4.50g, with an increase of 4.89–10.94% compared with *Hapl*-B2. For *Ppd-D1*, the HD of *Hapl*-D1 was about 2.52–5.98days earlier, with an advance range of 1.24–2.93%. *Hapl*-D1 reduced PH by about 14.43–27.41cm, with a decrease of 13.62–27.30%, compared with *Hapl*-D2. *Hapl*-D1 increased TGW by about 3.72–7.30g, with an increase of 11.12–21.45% compared with *Hapl*-D2. It is worth noting that *Ppd-B1* and *Ppd-D1* were significantly related to GW, a finding which was not observed for *Ppd-A1* (Figure 4E). *Hapl*-B1 and *Hapl*-D1 increased GW by about 0.09–0.13 and 0.15–0.28cm, respectively, with increases of 2.64–3.53 and 4.67–9.41%, compared with *Hapl*-B2 and *Hapl*-D2, respectively. However, the difference in GL between different haplotypes of *Ppd-1* was not significant (*p*>0.05; Figure 4F). Meanwhile, there was also no significant association between *Ppd-1* and the parameters SN, TNSS, and NGS (Figures 4G–I).

### *Ppd-1*-*Hapl*-A1, *Ppd-1*-*Hapl*-B1, and *Ppd-1*-*Hapl*-D1 Were Positively Selected for in Wheat Breeding

**Figure 5:**
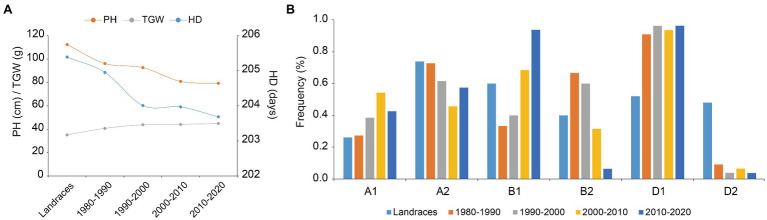
Favorable haplotypes of *Ppd-1* were selected for in wheat breeding programs. **(A)** Changes in PH, TGW, and HD in 188 accessions over decades in landraces and modern cultivars. In this population, 25 landraces are represented, as were 9, 26, 75, and 53 cultivars released in the 1980s, 1990s, 2000s, and 2010s, respectively. Data for PH, TGW, and HD are represented by the line chart using the BLUE mean under seven environments. **(B)** Frequency of *Ppd-A1*, *Ppd-B1*, and *Ppd-D1* haplotype changes over decades in landraces and modern cultivars.

To determine whether favorable haplotypes of *Ppd-1* were selected for during wheat breeding, we assessed the frequency changes of *Ppd-A1*, *Ppd-B1*, and *Ppd-D1* haplotypes in the 188 wheat accessions studied which originated over many decades in different regions of China (Supplementary Table S1). Based on phenotypic data exhibited in seven environments, PH declined from landraces to modern cultivars and fell continually in modern cultivars bred from the 1980s to the 2010s. TGW showed the opposite trend, increasing gradually from landraces to the modern cultivars. The heading date of modern cultivars advanced gradually with the increase in breeding years (Figure 5A). Compared with *Ppd-1-Hapl*-B2 and *Ppd-1-Hapl*-D2, *Hapl*-B1 and *Hapl*-D1 exhibited shorter PH, greater TGW, and earlier HD, which are all favorable haplotypes (Figures 4B–D). *Hapl*-A1 and *Hapl*-A2 exhibited no significant differences in PH, TGW, and HD, but *Hapl*-A1 was significantly superior to *Hapl*-A2 in terms of SL and TNSS (Figures 4A,H). Correspondingly, the frequencies of favorable haplotypes for *Ppd-A1*, *Ppd-B1*, and *Ppd-D1* gradually increased from 26.09, 60.00, and 52.00% in landraces, respectively, to 42.55, 93.62, and 96.23%, respectively, in the cultivars from the 2010s (Figure 5B), which indicated that *Ppd-1*-*Hapl*-A1, *Ppd-1*-*Hapl*-B1, and *Ppd-1*-*Hapl*-D1 were positively selected for during wheat breeding. It is worth noting that, for *Ppd-1*-*Hapl*-D1, there were sharp increases in frequencies from landraces to modern cultivars (Figure 5B), suggesting that selection occurred at the very beginning of modern wheat breeding programs. For *Ppd-A1* and *Ppd-B1*, the proportion of favorable haplotypes gradually increased over time.

### *Ppd-D1* Affects Rice Heading Date and Yield-Related Traits

**Figure 6:**
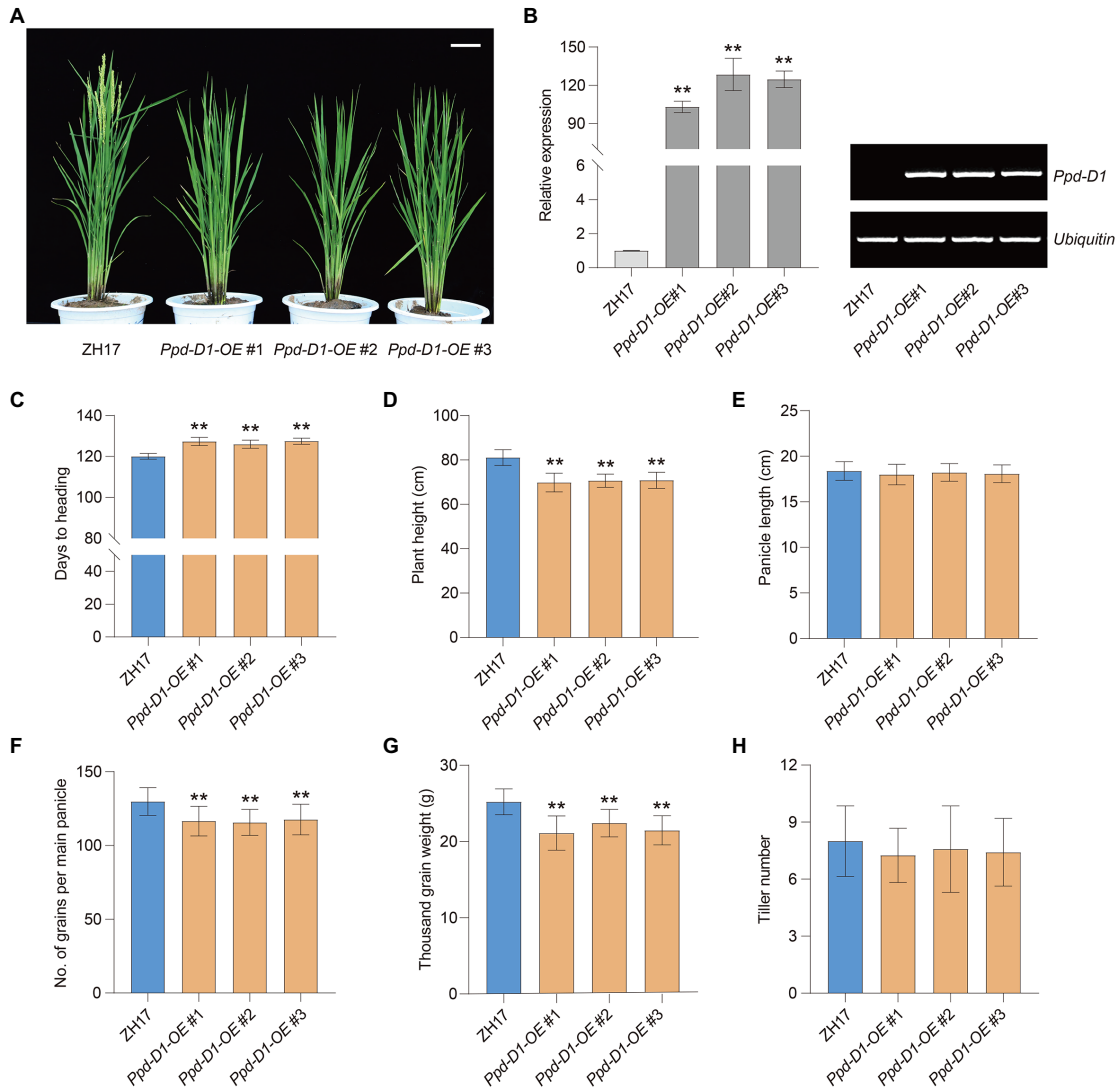
Overexpression of *Ppd-D1* affects rice yield-related traits. **(A)** Plant phenotypic comparison between wild-type Zhonghua 17 (ZH17) and *Ppd-D1-OE* transgenic lines. Scale bars=10cm. **(B)** qPCR (left) and RT-PCR (right) analysis of *Ppd-D1* expression in ZH17 and *Ppd-D1-OE* transgenic lines. The rice *Ubiquitin* gene was used as an internal control. Values are presented as mean±SD of three independent experiments. **(C–H)** Comparison of days to heading, plant height, panicle length, number of grains per main panicle, thousand-grain weight, and tiller number between ZH17 and *Ppd-D1-OE* transgenic lines (for each line, *n*=12). Values are presented as mean±SD. Phenotypic measurements of the transgenic plants were performed using three independent transgenic lines. Tukey’s test was performed between control and transgenic plants (^**^p<0.01).

To evaluate the effect of *Ppd-1* on yield-related traits, we introduced the overexpression construct (p*Ubi:Ppd-D1*, OE) into the *japonica* cultivar ZH17 (Figure 6A). Under field conditions, three representative homozygous lines overexpressing *Ppd-D1* (*Ppd-D1*-OE) were obtained for detailed analysis. Compared with ZH17, as the wild-type control, the *Ppd-D1-OE* transgenic plants showed significantly elevated *Ppd-D1* expression levels (Figure 6B). The heading date of *Ppd-D1-OE* transgenic lines was delayed by 5.92–7.42days (+4.93 to 6.18%), compared with wild-type plants (*p*<0.01; Figure 6C). Meanwhile, the *Ppd-D1-OE* transgenic lines showed reduced plant height (−12.64 to −13.87%) as well as decreased number of grains per main panicle (−9.44 to −10.91%) and thousand-grain weight (−11.08 to −16.24%; *p*<0.01; Figures 6D,F,G). However, there was no significant difference in either panicle length or tiller number (*p*>0.05; Figures 6E,H). Thus, the constitutive expression of *Ppd-D1* delayed the heading date and affected yield-related traits in rice.

### *Ppd-D1* Affects Grain Size and Quality in Rice

**Figure 7:**
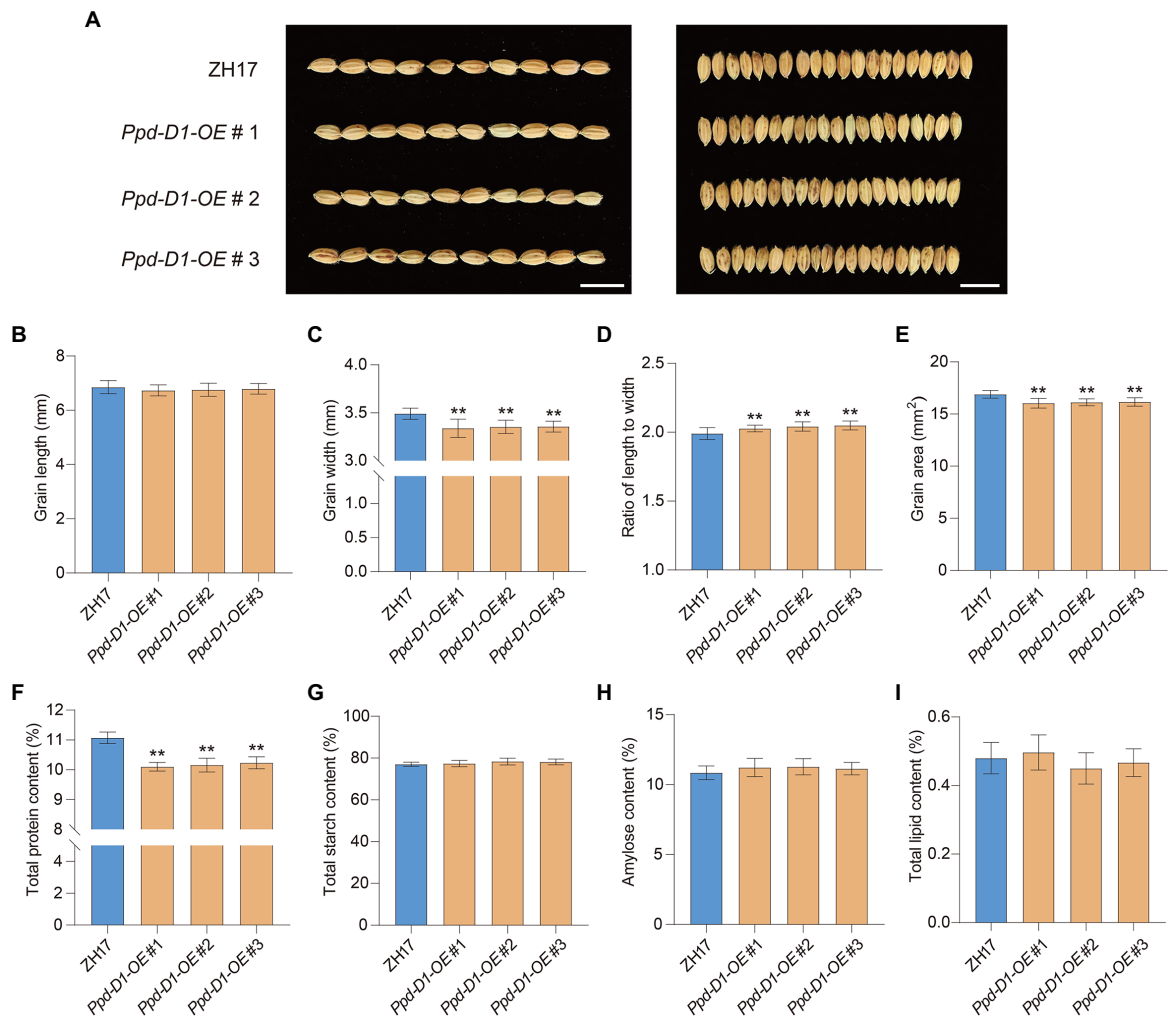
*Ppd-D1* affects grain size and quality in rice. **(A)** Comparison of grain size between Zhonghua 17 (ZH17) and *Ppd-D1-OE* transgenic lines. Scale bars=1cm. **(B–E)** Comparison of the grain length, grain width, the ratio of grain length to width, and grain area between ZH17 and *Ppd-D1-OE* (for each plant, *n*=50). **(F–I)** Comparison of total protein content, total starch content, total amylose content, and total lipid content between ZH17 and *Ppd-D1-OE* (for each line, *n*=5). Values are presented as mean±SD. Phenotypic measurements of the transgenic plants were performed using three independent transgenic lines. Tukey’s test was performed between control and transgenic plants (^**^p<0.01).

The functions of *Ppd-D1* during seed development were also evaluated (Figure 7A). Compared with ZH17, as the control, the *Ppd-D1-OE* transgenic lines showed decreased grain width (−3.87 to −4.37%), decreased grain area (−4.38 to −5.06%; Figures 7C,E), and increased ratio of length to width (+1.84 to 2.93%; *p*<0.01; Figure 7D). However, there was no significant difference in grain length (*p*>0.05; Figure 7B). We also tested the effect of *Ppd-D1* on the nutrient content of rice grains, including total protein content, starch content, amylose content, and total lipid content. The *Ppd-D1-OE* transgenic lines showed decreased total protein content (−7.56 to −8.79%), compared with the control wild-type plants (Figure 7F). However, there was no significant effect on total starch content, amylose content, and lipid content (*p*>0.05; Figures 7G–I). Thus, the constitutive expression of *Ppd-D1* affected grain size and seed quality in rice. These findings confirmed the potential application of *Ppd-D1* in improving crop grain traits.

## Discussion

### The *PRR* Family Members of the Circadian Clock Genes Play an Important Role in Crops

The circadian clock is the core part of the photoperiod regulatory system, and *PRR* is the key component of the circadian clock regulatory network. In addition to the discovery of the *PRR* family in the model plant *Arabidopsis thaliana*, which regulates the growth and development of plants and its responses to changes in the external environment, including stress, research into the circadian clock of crops has also gradually developed in recent years. *TaPRR1* is a core member of the wheat circadian clock. Sun et al. (2020) found that the expression of the *TaPRR1* gene was significantly correlated with yield-related traits and exhibited genetic variation and differentiation between landraces and modern cultivars. The wheat microRNA (tae-miR408)-mediated control of *TaPRR1* gene transcription is required for the regulation of heading date (Zhao et al., 2016). Circadian clock member *TaPRR73* affected heading date and plant height and promoted rice heading under long-day conditions (Zhang et al., 2016). The function of *PRR* gene family members in rice has also been reported. Studies have shown that rice *OsPRR37* may be involved in the regulation of *Hd3a* gene expression, thereby regulating the sensitivity of rice to photoperiod (Koo et al., 2013). The rice circadian clock system not only regulates the heading date of rice, but also participates in the tolerance response of rice to salt and cold stress (Xu et al., 2016; Wei et al., 2021). Studies have shown that OsPRR73 positively regulates rice salt tolerance by modulating *OsHKT2*;*1*-mediated sodium homeostasis (Wei et al., 2021).

### Epigenetic Modification of the Circadian System and Epigenetic Molecular Markers

The network architecture of the circadian system core oscillator is mainly composed of multiple core components through interlocked transcription–translation feedback loops. In addition to strict transcription and post-transcriptional regulation, their expression level and activity are also regulated by epigenetic modification. Studies have found that DNA methylation is an important epigenetic regulator for the precise maintenance of the plant circadian clock. Tian et al. (2021) proposed a new mechanism of DNA methylation controlled by the protein degradation cascade pathway SDC-ZTL-TOC1 to precisely regulate the clock pace, enriching our understanding of the regulation mechanism of the circadian system at the epigenetic level. Zhao et al. (2016) suggested that microRNAs might function in controlling the wheat heading date by mediating circadian clock gene expression, which provides important new information on the mechanism underlying heading date regulation in wheat.

Sun et al. (2014) found that DNA methylation occurs in the promoter region of the wheat photoperiod gene *Ppd-B1*, which affects gene expression level and subsequently wheat photoperiod response. It is worth noting that the hypermethylated region of the *Ppd-B1* gene overlaps with the deletion regions upstream of the *Ppd-A1* and *Ppd-D1* genes, implying that the upstream regulatory regions of *Ppd-A1*, *Ppd-B1*, and *Ppd-D1* have common key regulatory elements. The methylation level of *Ppd-B1* can be determined by bisulfite genomic sequencing, but this method is expensive. In the current paper, an epigenetic marker of *Ppd-B1* methylation has been developed, which can identify the *Ppd-B1* methylation level through restriction endonuclease digestion combined with PCR. Meanwhile, the high-methylation haplotype of *Ppd-B1* was shown to be positively selected in wheat breeding, and the development of molecular markers will be helpful in assisting wheat molecular breeding and genetic improvement.

### Research Prospects of the Crop Circadian Clock

In recent years, scientists have used the model plant *A. thaliana* to make progress in the study of the signal transduction mechanism of circadian clock-mediated plant growth and development, which has greatly promoted the development of this field. The functions of the circadian clock system in crops are diverse and conservative, but the mechanism of the circadian clock components in regulating the growth and development of crops still needs further study. The general impact of the circadian clock system on crops suggests that, by modifying the circadian rhythm, designing the timing of transgene expression and applying agricultural treatments at the most effective time of the day, future food production may be improved (Steed et al., 2021). Analyzing how the circadian clock system regulates the growth and development process of crops will hopefully illuminate the theoretical basis of chronobiology, provide high-quality genetic resources for crop molecular breeding, and increase crop yields (Wei et al., 2018).

*Photoperiod-1* (*TaPRR37*) encodes a member of the *PRR* protein family and is homologous to *Arabidopsis PRR7*. *AtPRR7* plays a role in clock function and photoperiod response, but the cereals genes may partially separate these functions, allowing mutations of the *PRR37* gene to manipulate photoperiod response without affecting clock function (Higgins et al., 2010). Therefore, the *Ppd-1* gene functions in the downstream of the circadian clock and is controlled by the output signal of the oscillator. However, in recent years, more and more reports suggested that *Ppd-1* is a member of the circadian clock. Steed et al. (2021) indicated that on the basis of its relationship to *Arabidopsis*, *PRR37* is assumed to have potential roles in the oscillator. Cao et al. (2021) described the PRRs, including the wheat *Ppd-1* gene, as a major component of the circadian clock. The function of *Ppd-1* in the oscillator needs to be further studied and elucidated in the future. As a member of the circadian clock system, *Ppd-1* has made a great contribution to the wheat “Green Revolution” (Borlaug, 1983). Making full use of the diversity of *Ppd-1* gene resources and developing simple and usable molecular markers are of great significance for the effective use of its tremendous and valuable allelic variation, thereby improving crop yields and quality.

## Data Availability Statement

The original contributions presented in the study are included in the article/Supplementary Material, further inquiries can be directed to the corresponding authors.

## Author Contributions

HS and FC designed and conceived the research. HS, YW, JL, GH, HXue, and HXu performed the experiments. HS and YW analyzed the data and wrote the paper. CZ and RQ supervised the project. All authors contributed to the article and approved the submitted version.

## Funding

This work was supported by the National Natural Science Foundation for Young Scholars of China (Grant No. 31801348), the Natural Science Foundation of Shandong Province, China (Grant No. ZR2019PC003), the National Natural Science Foundation of China (Grant Nos. 32072051 and 31871612), the Major Basic Research Project of Natural Science Foundation of Shandong Province, China (Grant No. ZR2019ZD16), the Youth Innovation Technology Support Planning Project for Institution of Higher Education of Shandong Province, China (Grant No. 2019KJF002), the Agricultural Variety Improvement Project of Shandong Province (2019LZGC016), and Yantai New and Old Kinetic Energy Conversion Research Institute and Yantai Science and Technology Achievement Transfer Demonstration Base Funded Project (2019XJDN007).

## Conflict of Interest

The authors declare that the research was conducted in the absence of any commercial or financial relationships that could be construed as a potential conflict of interest.

## Publisher’s Note

All claims expressed in this article are solely those of the authors and do not necessarily represent those of their affiliated organizations, or those of the publisher, the editors and the reviewers. Any product that may be evaluated in this article, or claim that may be made by its manufacturer, is not guaranteed or endorsed by the publisher.
