# Supplementary figures and images for: Functional Analysis of the “Green Revolution” Gene Photoperiod-1 and Its Selection Trends During Bread Wheat Breeding
Source: Front Plant Sci. 2021 Nov 11;12:745411. doi: 10.3389/fpls.2021.745411 (PMC8631784; doi:10.3389/fpls.2021.745411)

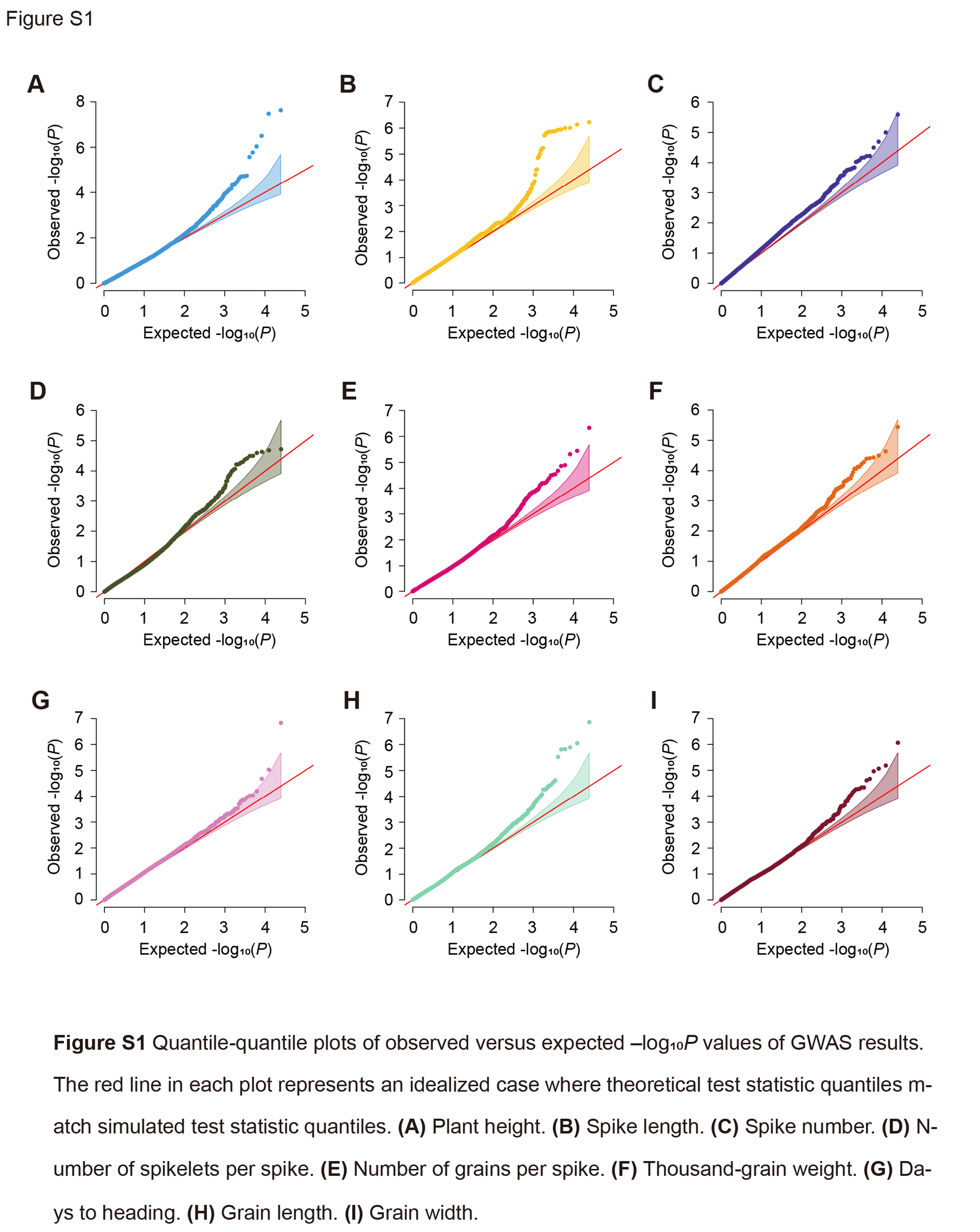

Supplement: Supplementary file 2 [file Image_1.TIF]
